# Integrated bioinformatics analysis identifies established and novel TGFβ1-regulated genes modulated by anti-fibrotic drugs

**DOI:** 10.1038/s41598-022-07151-1

**Published:** 2022-02-23

**Authors:** Ava C. Wilson, Joe Chiles, Shah Ashish, Diptiman Chanda, Preeti L. Kumar, James A. Mobley, Enid R. Neptune, Victor J. Thannickal, Merry-Lynn N. McDonald

**Affiliations:** 1 Department of Epidemiology, School of Public Health, University of Alabama at Birmingham, Birmingham, AL USA; 2 Division of Pulmonary, Allergy and Critical Care Medicine, Department of Medicine, University of Alabama at Birmingham, Birmingham, AL USA; 3 Department of Orthopedic Surgery, University of Alabama at Birmingham, Birmingham, AL USA; 4 Division of Molecular and Translational Biomedicine, Department of Anesthesiology and Perioperative Medicine, University of Alabama at Birmingham, Birmingham, AL USA; 5 Department of Medicine, Johns Hopkins University, Baltimore, MD USA; 6 John W. Deming Department of Medicine, Tulane University School of Medicine, New Orleans, LA USA; 7 Department of Genetics, University of Alabama at Birmingham, Birmingham, AL USA

**Keywords:** Computational biology and bioinformatics, Respiratory tract diseases

## Abstract

Fibrosis is a leading cause of morbidity and mortality worldwide. Although fibrosis may involve different organ systems, transforming growth factor-β (TGFβ) has been established as a master regulator of fibrosis across organs. Pirfenidone and Nintedanib are the only currently-approved drugs to treat fibrosis, specifically idiopathic pulmonary fibrosis, but their mechanisms of action remain poorly understood. To identify novel drug targets and uncover potential mechanisms by which these drugs attenuate fibrosis, we performed an integrative ‘omics analysis of transcriptomic and proteomic responses to TGFβ1-stimulated lung fibroblasts. Significant findings were annotated as associated with pirfenidone and nintedanib treatment in silico via Coremine. Integrative ‘omics identified a co-expressed transcriptomic and proteomic module significantly correlated with TGFβ1 treatment that was enriched (FDR-p = 0.04) with genes associated with pirfenidone and nintedanib treatment. While a subset of genes in this module have been implicated in fibrogenesis, several novel TGFβ1 signaling targets were identified. Specifically, four genes (*BASP1, HSD17B6, CDH11*, and *TNS1*) have been associated with pirfenidone, while five genes (*CLINT1, CADM1, MTDH, SYDE1*, and *MCTS1*) have been associated with nintedanib, and *MYDGF* has been implicated with treatment using both drugs. Using the Clue Drug Repurposing Hub, succinic acid was highlighted as a metabolite regulated by the protein encoded by *HSD17B6***.** This study provides new insights into the anti-fibrotic actions of pirfenidone and nintedanib and identifies novel targets for future mechanistic studies.

## Introduction

Affecting all organs and contributing to numerous diseases, fibrosis is a leading cause of mortality and morbidity, accounting for up to 45% of all deaths globally^1^. Fibrosis is caused by abnormal extracellular matrix (ECM) deposition by activated (myo)fibroblasts that results in scarring of organ-specific tissues^2–4^. Transforming growth factor beta 1 (TGFβ1) plays a central role in fibrogenesis, primarily by inducing the transition of fibroblasts into myofibroblasts^5^. New candidates for treating fibrotic diseases are needed as only two drugs (pirfenidone and nintedanib) have received regulatory approval to treat fibrosis, specifically idiopathic pulmonary fibrosis (IPF)^6^. IPF is an age-related interstitial pulmonary disease characterized by worsening dyspnea and reduced lung function due to progressive, irreversible fibrosis^7,8^. In addition to a poor quality of life, the prognosis for IPF patients is poor with a median survival time of 3 years after diagnosis^9^. Although both pirfenidone and nintedanib are widely used in the treatment of IPF the underlying mechanisms of their biological action(s) remains poorly understood^10–12^. Further, pirfenidone and nintedanib have varying efficacy in treating IPF necessitating a deeper understanding of their mechanisms in delaying disease progression^13,14^.

Currently, there is limited data regarding the molecular mechanisms of pirfenidone and nintedanib on the canonical TGFβ1 pathway. Reports of the potential anti-fibrotic actions of pirfenidone have been limited to candidate gene analyses^15^. The efficacy of pirfenidone in treating fibrosis has been attributed to pleiotropic modes of action including anti-fibrotic, anti-inflammatory, and antioxidant effects^16^. Ballester et al. found pirfenidone’s inhibitory effect on TGFβ1-induced fibrosis is mediated through various mechanisms including the inhibition of transmembrane mucin 1 c-terminal cytoplasmic tail (MUC1-CT) phosphorylation, β-catenin activation, nuclear complex formation of the phospho-SMAD3/MUC1-CT/active-β-catenin complex, and SMAD-binding element (SBE) activation^15^. Nintedanib in a tyrosine kinase inhibitor that inhibits multiple tyrosine kinase signaling pathways including VEGF, PDGF, and FGF^14^. Both drugs have pleiotropic effects targeting different fibrotic mechanisms, however, multi-omics studies investigating the antifibrotic mechanism of pirfenidone and nintedanib in relation to TGFβ1 signaling responses are lacking. Although both drugs slow disease progression, neither drug improves or stabilizes lung function, nor improves quality of life^17^. In addition, both drugs have tolerability issues, facilitating the need for the identification of novel IPF drug targets^17^.

To begin to address these gaps in our understanding of fibrotic disease development and treatment, we hypothesized that identifying TGFβ1 induced transcriptomic and proteomic changes that are co-expressed with genes associated with response to pirfenidone and nintedanib may identify novel mechanisms of drug action, and potentially uncover new targets for future anti-fibrotic therapies. To test this hypothesis, transcriptomic and proteomic profiles with and without TGFβ1 treatment of human fetal lung mesenchymal cells (IMR-90) were analyzed. TGFβ1-induced changes integrated across the transcriptome and proteome were identified at the single transcript and protein and network levels using Weighted Gene Co-Expression Network Analysis (WGCNA) software^18^. Further, we provide a robust annotation of integrated transcriptomic and proteomic signals significantly correlated with TGFβ1 treatment, including mining of the literature for pirfenidone and nintedanib associated genes, mining of known functional pathways to identify TGFβ1 signaling targets, screening of drug repurposing metadata, and pathway analyses.

## Results

### Differentially expressed genes and proteins induced by TGFβ1

At the single gene level, TGFβ1 induced differential expression of 780 genes (Table S1). Among these, 416 genes were upregulated, and 364 genes were downregulated (Table S1). Collectively, the 780 TGFβ1 differentially expressed genes were enriched with genes involved in collagen deposition in the extracellular matrix (ECM) (GO Collagen Containing Extracellular Matrix, FDR p-value = 3.84 × 10^–34^), in addition to genes involved with apoptosis (GO Apoptotic Process, FDR p-value = 1.89 × 10^–43^), extracellular signaling (GO Enzyme Linked Receptor Protein Signaling Pathway, FDR p-value = 1.22 × 10^–38^), and genes involved in the epithelial mesenchymal transition (EMT) in wound healing and fibrosis (Hallmark Epithelial Mesenchymal Transition, FDR p-value = 3.31 × 10^–79^) (Table S2).

At the single protein level, TGFβ1 significantly altered the levels of eight proteins (Table S3). Of these proteins, TGFβ1 stimulated an increase for six proteins (PDZ and LIM domain protein 5, Calponin 1, Collagen alpha-1 V chain, Tensin 1, Calponin 3, and LIM domain and actin binding protein 1) and a decrease for two proteins (Calpain 2 catalytic subunit and Collagen alpha-1 IV chain 1) (Table S3). Collectively this set of TGFβ1 altered proteins is enriched with those encoding ECM proteoglycans (REACTOME ECM Proteoglycans, FDR p-value = 0.032), collagen biosynthesis and modifying enzymes (REACTOME Collagen Biosynthesis and Modifying Enzymes, FDR p-value = 0.0281), and genes up-regulated by TGFβ1 (McBryan Pubertal TGF-β1 Targets Up, FDR p-value = 0.00447) (Table S4).

### TGFβ1 induced co-expression of transcriptomic modules

**Figure 1:**
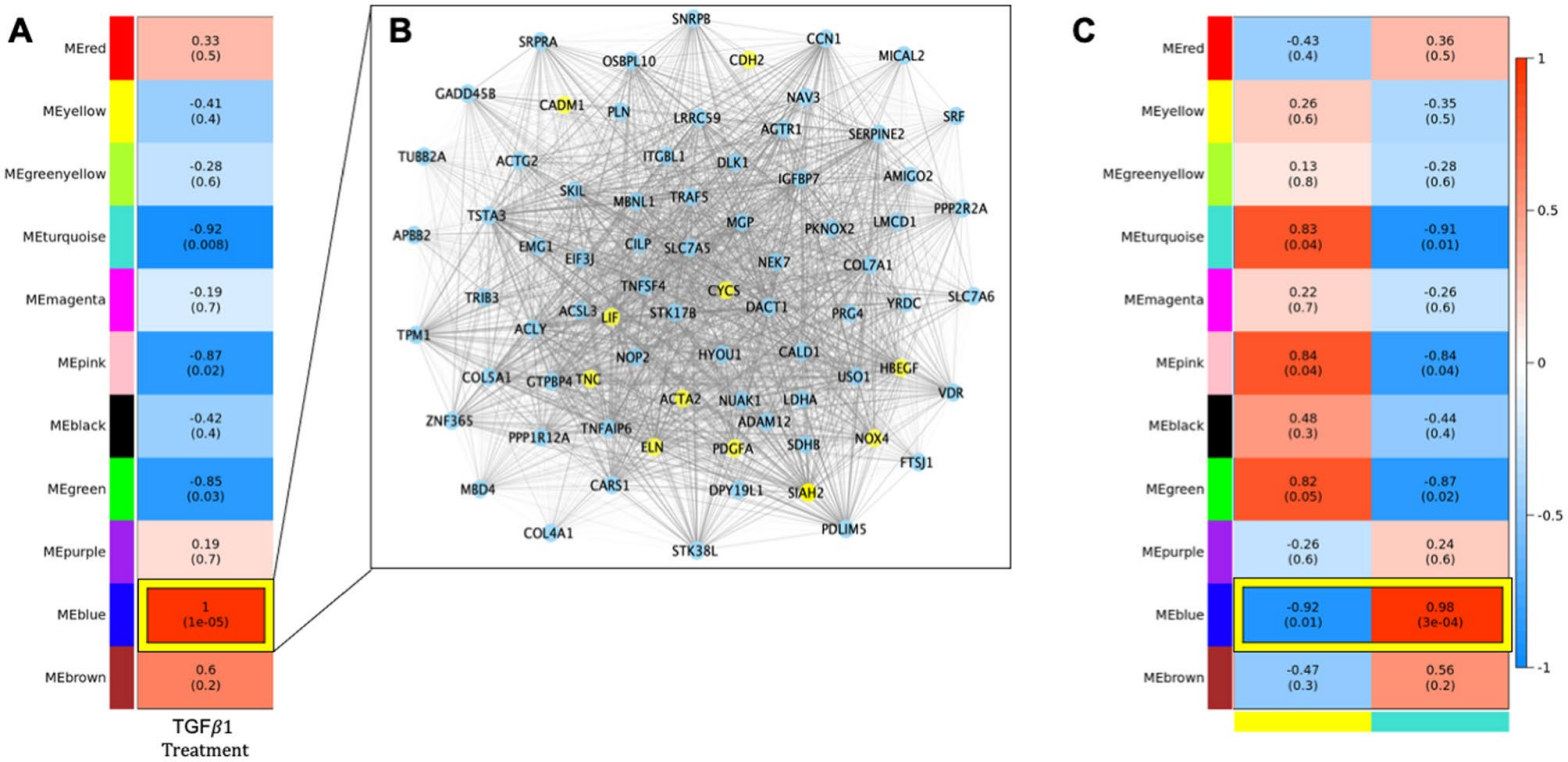
Results of integrative ‘omics analysis of proteomic and transcriptomic data generated from IMR-90 cells with and without TGFβ1 treatment. (**A**) Transcriptomic module association with TGFβ1 treatment: Values in each cell represent correlation, in parentheses, with p-values between each module of co-expressed transcripts and TGFβ1 treatment. Heatmap shading corresponds to strength of association where darker red cells have higher upregulation and darker blue cells have higher downregulation based on correlation. Cells outlined in yellow withstand Bonferroni correction for multiple testing based on the number of modules generated. (**B**) Network visualization of hub genes in the blue transcriptomic module. Genes with a kME larger than 0.99 were selected for visualization in the blue module. The thickness of the edge corresponds to increasing topological overlap (TOM), a measure of the strength of correlation between transcript levels, which is the Pearson’s correlation obtained from the adjacency matrix. Nodes labeled in yellow correspond to single genes in the blue module that are annotated as associated with pirfenidone and/or nintedanib treatment. (**C**) Results from integration of transcriptomic and proteomic data. Values in each cell represent correlation, p-values in parentheses, between each module of co-expressed transcripts with TGFβ1 treatment and modules of co-expressed proteins. The y-axis corresponds to transcriptomic modules generated using WGCNA. The x-axis corresponds to the yellow and turquoise proteomic modules. Individually, the yellow and turquoise proteomic modules were significantly correlated with TGFβ1 treatment (depicted in Fig. 2).

At the network level, weighted gene co-expression analyses identified 11 modules of co-expressed genes. TGFβ1 induced significant upregulation of the 1564 blue module transcripts (r_blue_ = 1.0, p_blue_ = 1 × 10^–5^, Fig. 1A, Table S5). In the blue transcriptomics module, 677 were novel genes not previously implicated in TGFβ1 signaling (Table S5). The mean and standard deviation of the kME for the blue module was 0.87 ± 0.14 with 5.3% of the transcripts meeting the hub criteria of kME > 0.99 (Table S6). Visualization of the connectivity of the blue module hub genes is depicted in Fig. 1B.

### Network analyses indicates two proteomic modules correlated with TGFβ1 treatment

**Figure 2:**
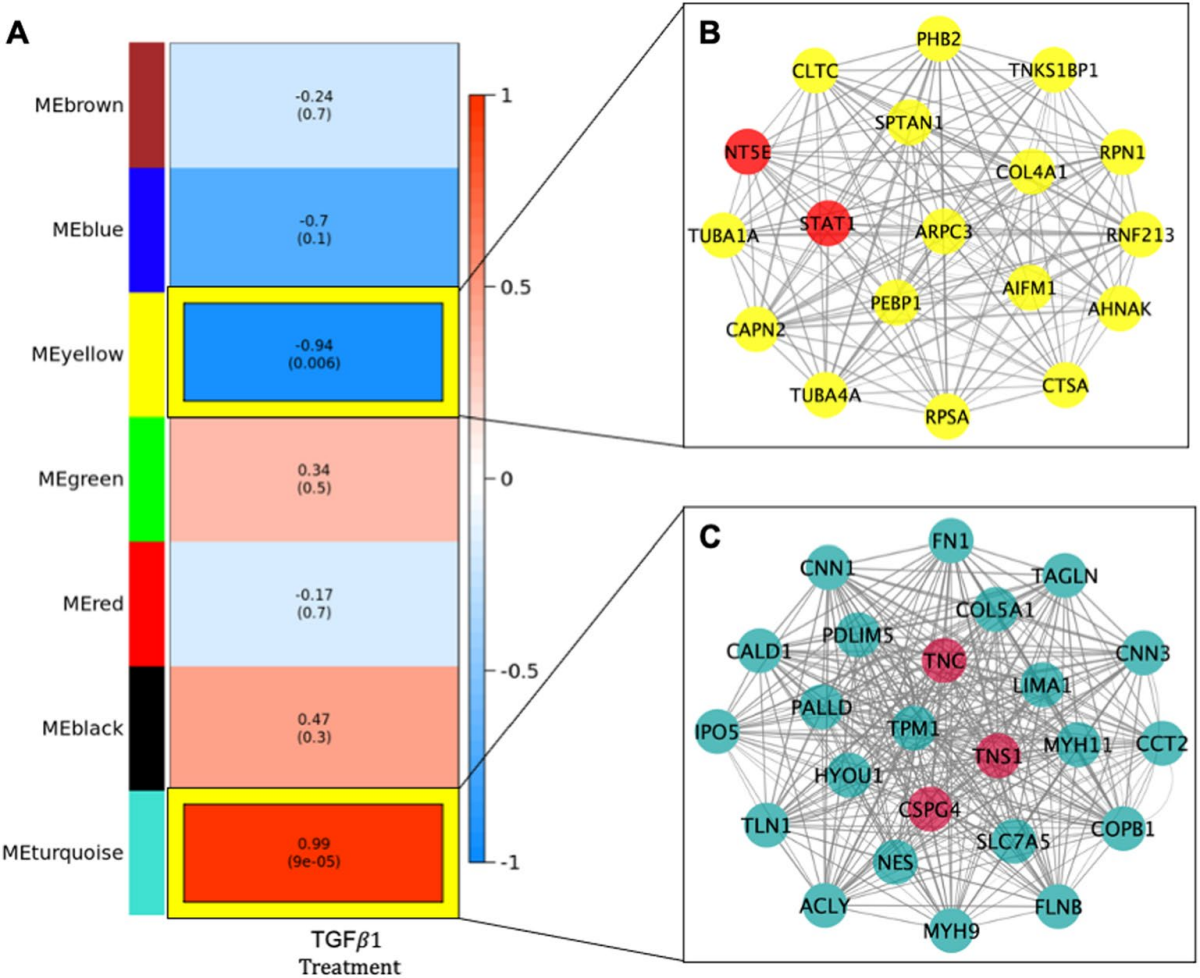
Weighted gene co-expression network analysis of proteomic data generated from IMR-90 cells with and without TGFβ treatment. (**A**) Proteomic Modules Associated with TGFB1 Treatment in IMR-90 cells. Values represent correlation with p-values in parentheses between each module and trait. Heatmap shading corresponds to strength of association where darker red cells have higher upregulation and darker blue cells have higher downregulation based on correlation. Text outlined in yellow denotes result withstands Bonferroni correction for multiple testing based on the number of modules generated. (**B**,**C**) Network of hub proteins in proteomic modules significantly associated with TGFβ Treatment. Proteins with a kME larger than 0.90 were selected for visualization in the turquoise (**B**) and yellow (**C**) modules. The size of the circle in each network corresponds to increasing module membership and the thickness of the edge corresponds to increasing topological overlap (TOM), a measure of the strength of correlation between protein levels, which is the Pearson’s correlation obtained from the adjacency matrix. Yellow nodes correspond to significant single proteins in the turquoise module associated with TGFβ1 treatment. Red nodes correspond to known targets of pirfenidone and/or nintedanib.

At the network level, the weighted gene co-expression protein network consisted of 7 modules. Of which, the yellow and turquoise modules were significantly correlated with TGFβ1 treatment at levels withstanding Bonferroni correction (Fig. 2A). Collectively, the 78 proteins in the yellow module (r_yellow_ = − 0.94, p_yellow_ = 0.006) were significantly downregulated with TGFβ1 treatment (Fig. 2A). In the turquoise module, 115 proteins (r_turquoise_ = 0.99, p_turquoise_ = 9 × 10^–5^) module were collectively upregulated with TGFβ1 treatment (Fig. 2A). The mean and standard deviation of the kME for the yellow and turquoise protein modules were 0.78 + 0.13 and 0.73 + 0.18, respectively (Table S6). Additionally, 23.1% and 21.7% of the proteins in the yellow and turquoise modules, respectively, were hub proteins (Table S6). Visualization of the connectivity of hub proteins, kME > 0.9, in the yellow (N = 18) and turquoise (N = 25) modules is depicted in Fig. 2B,C. Of the 78 proteins in the yellow module, 26 were identified as novel proteins involved in TGFβ1 signaling, with 2 proteins (*LGALS1* and *COL6A2*) known to be secreted proteins (Table S5). Of the 115 proteins in the turquoise module, 43 were identified as novel proteins involved in TGFβ1 signaling, with 5 proteins (*COPA, TGFBI, COL5A2, PXDN,* and *COL5A1*) known to be secreted (Table S5).

### Integrative ‘omics analyses identifies co-expressed transcript and peptides modules that correlate with TGFβ1 treatment

The blue transcriptomics module was correlated with the turquoise and yellow proteomic modules (Fig. 1C). The expression of genes in the blue module was positively correlated with the proteins in the turquoise module (Fig. 1C: r = 0.98, p = 3 × 10^–4^). Whereas the expression of genes in the blue module genes was negatively correlated with protein levels in the yellow module but at a nominal (p < 0.05) level (Fig. 1C: r = − 0.92, p = 0.01). Several of the TGFβ1 significantly differentially expressed genes (Table S5) had encoded proteins which were hub members of the yellow (*COL4A1)* and turquoise (*CNN1, TNS1, COL5A1,* and *PDLIM5)* proteomics module (Fig. 2B,C). The five genes and proteins were enriched for pro-fibrotic pathways including collagen formation (REACTOME collagen formation, FDR p-value = 0.021), ECM proteoglycans (REACTOME ECM proteoglycans, FDR p-value = 0.017), and PDGF signaling (REACTOME PDGF Signaling, FDR p-value = 0.016), among others.

### Novel fibrotic biomarkers common to pirfenidone, nintedanib, and TGFβ1 signaling

Of the genes annotated as response to pirfenidone and/ or nintedanib treatment, 264 genes were specific to pirfenidone and 150 genes were specific to nintedanib. A total of 149 genes were identified as being associated with both pirfenidone and nintedanib treatment (Table S8). The blue module was significantly enriched (FDR-p = 0.0395) for transcripts from genes associated with both pirfenidone and nintedanib treatment. Among these, 4 genes (*BASP1, HSD17B6, CDH11*, and *TNS1*) associated with pirfenidone were identified as novel genes not found in known pathways containing TGFβ1 (Table 1). Additionally, 5 genes (*CLINT1, CADM1, MTDH, SYDE1*, and *MCTS1*) associated with nintedanib treatment have not previously been classified as involved with TGFβ1 signaling (Table 1). One gene, *MYDGF*, was highlighted as being associated with both pirfenidone and nintedanib and not a member of known TGFβ1 signaling pathways (Table 1).

**Table 1:** Novel genes targeting pirfenidone, nintedanib, or both pirfenidone and nintedanib.

Gene name	Gene	Blue module membership
**Pirfenidone only**		
Brain abundant membrane attached signal protein 1	*BASP1*	0.76
Hydroxysteroid 17-beta dehydrogenase 6	*HSD17B6*	0.99
Cadherin 11	*CDH11*	0.92
Tensin 1	*TNS1*	0.93
**Nintedanib only**		
Clathrin interactor 1	*CLINT1*	0.97
Cell adhesion molecule 1	*CADM1*	0.95
Metadherin	*MTDH*	0.90
Synapse defective rho GTPase homolog 1	*SYDE1*	0.70
MCTS1 re-initiation and release factor	*MCTS1*	0.62
**Pirfenidone and nintedanib**		
Myeloid derived growth factor	*MYDGF*	0.94

Novel is defined as not previously identified in known TGFβ1 signaling pathways.

### Pathway analysis of novel fibrotic biomarkers in context with known pathobiology

The genes in the blue module annotated as associated with pirfenidone are enriched for genes involved in the epithelial mesenchymal transition (Hallmark Epithelial Mesenchymal Transition, FDR p-value = 1.1 × 10^–9^), tissue morphogenesis (GO Tissue Morphogenesis, FDR p-value = 3.2 × 10^–5^), and the inflammatory response (Hallmark Inflammatory Response, FDR p-value = 9.6 × 10^–5^), among others (Table S9). The genes in the blue module annotated as associated with nintedanib were enriched for genes involved in the response to wounding (GO Response to Wounding, FDR p-value = 2.2 × 10^–2^), apoptosis (Hallmark Apoptosis, FDR p-value = 9.2 × 10^–3^), and platelet derived growth factor binding (GO Platelet Derived Growth Factor Binding, FDR p-value = 4.7 × 10^–3^) (Table S9). Genes annotated as associated with both pirfenidone and nintedanib in the blue module were enriched with members of the collagen containing extracellular matrix (GO Collagen Containing Extracellular Matrix, FDR p-value = 1.8 × 10^–12^), those involved in mesenchymal cell differentiation (GO Mesenchymal Cell Differentiation, FDR p-value = 3.2 × 10^–9^), and lung fibrosis (WP Lung Fibrosis, 1.8 × 10^–6^), among others (Table S9).

### Evidence for drug repurposing based on known drug targets of novel TGFβ1 genes

Using the Clue Drug Repurposing Hub, succinic acid was highlighted as a metabolite regulated by the protein encoded by *HSD17B6* (Hydroxysteroid 17-Beta Dehydrogenase 6) (Table S10)^19^.

## Discussion

In this study, we provide new insights into the anti-fibrotic mechanisms of pirfenidone and nintedanib and uncover novel drug targets in TGFβ1-driven fibrosis. Using an integrative ‘omics approach, we identified modules of co-expressed transcripts and proteins induced by TGFβ1 in fibroblasts, key effector cells of tissue/organ fibrosis. We used an agnostic approach to identify markers of early TGFβ1-induced fibrogenesis and found TGFβ1 induced genes annotated as associated with pirfenidone and nintedanib treatment. In particular, we demonstrate evidence for a common mechanism of action for pirfenidone and nintedanib in modulating TGFβ1-induced *MYDGF*. Pirfenidone and nintedanib may mediate their anti-fibrotic effects via different mechanisms, such as *BASP1, HSD17B6, CDH11*, and *TNS1* with pirfenidone, and *CLINT1, CADM1, MTDH, SYDE1*, and *MCTS* with nintedanib*.*

Both pirfenidone and nintedanib likely influence the expression of several genes regulated by TGFβ1. Genes annotated as associated with pirfenidone treatment included *BASP1, HSD17B6, CDH11*, and *TNS1*. While *BASP1* and *HSD17B6* have been associated with multiple cancers, both *CDH11* and *TNS1* have been linked to mesenchymal activation and pulmonary fibrosis^20–24^. The cadherin 11 protein, encoded by *CDH11*, is a member of a family of integral membrane proteins responsible for the mediation of calcium-dependent cell to cell adhesion and has been implicated in epithelial–mesenchymal transition and pulmonary fibrosis^23^. Tensin 1, encoded by *TNS1*, is a key protein that is a component of fibrillar adhesions that attach to the extracellular matrix and is essential for myofibroblast differentiation^24^. This may indicate the clinical efficacy of pirfenidone and nintedanib in patients with fibrotic lung disease may involve both shared (common) pathways and single genes that mediate pro-fibrotic effects. The multifunctional effects of these drugs may also explain the need for combinatorial therapeutic approaches or single agents with pleiotropic effects. We found *CLINT1, CADM1, MTDH, SYDE1*, and *MCTS1* are annotated as being associated with nintedanib treatment; of these, *CADM1* and *MTDH* have been implicated in fibrosis. *CADM1*, an immunoglobulin superfamily member, has been reported to contribute pro-fibrotic effects through direct effects on fibroblasts and indirect effects on mast cell adhesion^25,26^. Metadherin (*MTDH*) has been shown to mediate changes consistent with epithelial–mesenchymal transition in kidney fibrosis^27^.

In our study, *HSD17B6* (Hydroxysteroid 17-Beta Dehydrogenase 6) was identified as a member of the highly TGFβ1 correlated blue module and as a single gene significantly upregulated (log FC = 3.48, FDR *p*-value = 0.0021) in TGFβ1 treated cells. *HSD17B6* has oxidoreductase activity and plays a key role in androgen catabolism and previous studies have demonstrated an association between androgen deficiency and cavernosal fibrosis^28,29^. In our in-silico analyses, *HSD17B6* was annotated as associated with pirfenidone. Previous studies have identified *GLRX*, also having oxidoreductase activity, as a therapeutic target of pirfenidone whose forced expression was sufficient to inhibit or reverse liver fibrosis^30^. Interestingly, single cell RNA-seq demonstrated *HSD17B6* as highly expressed in mesothelial cells in IPF^31^. Mesothelial cells play a direct role in fibrogenesis, and antioxidants have been shown to alleviate TGFβ1 induced EMT in mesothelial cells^32,33^. Using the Clue Drug Repurposing Hub, we were able to identify succinic acid as a likely target of the protein encoded by *HSD17B6.* Increased accumulation of succinate has been implicated as a promoter of the development of fibrosis in the lung and liver^34,35^. Interestingly, succinic acid has been identified as an important signaling molecule in both pulmonary and liver fibrosis with the development of therapies targeting succinate proposed as a potential treatment to prevent and/or cure fibrosis in these tissues^34,35^. Although succinate may have more affinity for its receptor (succinate receptor 1), its expression is varied according to tissue type^36^. However, compared to other tissues in the body, including liver tissue, the expression of succinate receptor 1 in the lung is low^36^. This suggests that succinate may mediate biological effects independent of its receptor(s) activation, such as post-translation modifications of proteins involving succinylation^37^.

*MYDGF* (also known as IL-25), which encodes the Myeloid Derived Growth Factor protein, was co-expressed with genes up or down regulated in TGFβ1 induced model of fibrosis. To our knowledge, this is first time *MYDGF,* annotated as associated with pirfenidone and nintedanib treatment, has been shown to be regulated by TGFβ1^38^. The biological function of *MYDGF* is relatively unknown; however, previous studies in mouse models of coronary artery disease have demonstrated that monocytes and macrophages secrete *MYDGF* as a protective and reparative response following myocardial infarction^39^. *MYDGF* treatment was shown to reduce scar size and contractile dysfunction and has been identified as a potential therapeutic target for cardiac fibrosis^39,40^. Although an agnostic approach was employed to identify *MYDGF* as a novel TGFβ1 signaling target, it was co-expressed with other known TGFβ1 signaling targets (*VEGF* and *ACTA2*, among others), providing additional support for this finding^41^.

Our study has several strengths and limitations. A strength of our study is that it presents the first integrated proteomic and transcriptomic network analysis of human lung fibroblasts treated with TGFβ1. We also took advantage of a network-based approach which highlighted new protein and gene expression modules associated with TGFβ1 treatment. Through the integration of significant proteomic modules into the transcriptomic module-trait correlation, we were able to systematically investigate the interplay of downstream targets associated with TGFβ1. Further, PSEA and GSEA indicated proteins and genes in our agnostically derived modules were significantly enriched for pathways with known relevant to TGFβ1 biology. Among the limitations, we analyzed proteomic and transcriptomic data generated at a single time point. A longitudinal analysis would have provided a deeper understanding of the changes in protein levels and gene expression over time. However, by integrating our proteomic and transcriptomic results, we were able to observe the downstream mechanisms contributing to fibrogenesis. Further, we limited our studies to exploring primary effects of TGFβ1 signaling in fibroblasts obtained from a cell line as opposed to primary cells. Another limitation of this study is the lack of an equivalent study in which we could bioinformatically validate the genes annotated as associated with pirfenidone and nintedanib not found in known pathways containing TGFβ1. However, these findings have the potential to identify novel pathways and/or molecular targets within these pathways due to the agnostic approach employed by this study. Follow-up via replication is an important future direction for our understanding of the robustness of our novel finding. Further, unlike transcripts, proteins cannot be amplified, and as this was not a targeted study, but rather a global discovery proteomics analysis, we are limited to what is observed. However, with the use of systems analysis combined with transcriptomics, this data pinpoints key pathways and molecular changes with high confidence; therefore, the lack of observation of a few known proteins should not detract from the utility of this kind of multi-Omics study. Finally, the genes associated with pirfenidone and nintedanib obtained from Coremine may have data not just from IMR-90 cells, but also from other cell lines that may confound the results.

In conclusion, we identified potential molecular targets involved in TGFβ1 signaling driving myofibroblast differentiation and preclinical fibrosis development. We also demonstrated the utility of integrative network analyses to identify novel molecular targets and pathways that help elucidate the role of TGFβ1 signaling in the anti-fibrotic actions of pirfenidone and nintedanib. Further studies of these novel targets are warranted to confirm reproducibility and potential therapeutic efficacy in relevant pre-clinical models.

## Methods

### Study design and ethics

IRB approval was obtained from the University of Alabama at Birmingham for analyses presented in this manuscript. All statistical analyses, including WGCNA, were performed in R, version 3.6.0. Cytoscape, version 3.8.1, was used to visualize molecular pathways of interest.

### Cell culture

Human fetal lung mesenchymal cells (hFLMCs; IMR-90 cells) were obtain from Coriell Cell Repositories, Institute for Medical Research, Camden, NJ. IMR-90 cells were cultured in DMEM (Life Technologies, Inc.) supplemented with 10% fetal calf serum (Hyclone Laboratories, Logan, UT), 100 U/ml penicillin, 100 µg/ml streptomycin, and 1.25 µg/ml amphotericin B. IMR-90 cells were incubated at 37 °C in 5% CO_2_ and 95% air. For both transcriptomic and proteomic analyses, a total of 6 flasks of IMR-90 cells with (N = 3) replicates per group, TGFβ treatment (2 ng/ml for 16 h) and no TGFβ treatment, were included in this study.

### Transcriptomic data

Gene expression data was obtained from GEO (GEO: GSE17518). Sample preparation and quality control for gene expression data have been previously described^42^. Briefly, gene expression data was generated from IMR-90 cells and profiled using the Affymetrix Human U133A array (Fig. S1). For (N = 3) samples treated with TGFβ, mRNA was collected 48 h post treatment^42,43^. Gene expression data was normalized using log transformation. Probes with low variance and those which did not annotate within a specific gene were removed, leaving a final sample size of 6,456 probes for analysis. The amount of missing data for each probe was assessed for quality control, and no probes were removed due to missingness, defined as missing in more than 1 of either control or treatment samples (Fig. S1).

### Proteomic data

Proteomics analyses were carried out as previously referenced with minor changes (Ludwig et. al, under section 2.5 nLC-ESI-MS2 under Protein IDs for GeLC)^44^. The protein fractions were quantified, 40 µg of protein per sample were reduced with DTT and denatured at 70 °C for 35 min prior to loading onto 10% Bis–Tris Protein gels and separated. The gels were stained overnight with colloidal Coomassie for visualization purposes, the entire gel lane was cut into 6-MW fractions, and each plug was equilibrated in 100 mM ammonium bicarbonate (AmBc), and digested overnight with Trypsin Gold, Mass Spectrometry Grade (Promega, Cat.# V5280) following manufacturer’s instruction. Peptide extracts were reconstituted in 0.1% Formic Acid/ ddH_2_O at 0.1 µg/µL. Mass spectrometry was carried out, and the data was processed, searched, filtered, grouped, and quantified, as previously reported in detail^45^. Following protein identification, and relative quantification by normalized spectral counting (NSC), the most statistically significant changed proteins from each pairwise comparison were analyzed^45^.

An overview of the computational integrated ‘omics analysis methods used are provided in Fig. S1. Peptides missing in more than 1 of either control or treatment samples were removed from the analysis. Proteomic data was normalized using a log transformation and proteins with low variance were removed from analyses, leaving a final sample size of N = 533 peptides for analyses (Fig. S1). The R MICE package was used to impute missing data^46^.

### Single transcript analysis

Linear regression models were fit for each single-gene transcript using an empirical Bayes method to determine if any significant single-gene transcripts were associated with TGFβ1 treatment. False discovery rate (FDR) p-values were calculated based on the number of transcripts in the array (N = 22,284). In instances were multiple probes mapping to a single gene, the probe with the highest mean expression value was selected^47^.

### Single peptide analysis

Linear regression models were fit for each protein using an empirical Bayes method to determine if any significant proteins were associated with TGFβ1 treatment. False discovery rate (FDR) p-values were calculated based on the number of proteins identified from the column post-quality control (N = 533).

### Weighted gene co-expression (WGCNA) analysis

WGCNA^18^ is an established network analysis method which maximizes the statistical power of complex analyses by taking into account the correlated nature of biological networks. WGCNA can be used to translate and integrate ‘omics data into networks of co-expressed biomarkers which can be tested for association with phenotypes. Focusing on networks as opposed to single, candidate biomarkers provide a biologically relevant approach to visualizing ‘omic pathways through the observation of combined influence and interrelation of multiple molecular layers on the disease process. In addition, WGCNA networks are generated using an agnostic approach as opposed to reliance on known biological pathways to identify a wider scope of novel biomarkers. The WGCNA R package^18^ was used to identify modules of co-expressed proteins and genes (also termed eigenprotein and eigengene), which consist of groups of proteins and genes with similar protein and gene expression patterns. A signed correlation network was built using a Pearson’s correlation with a soft thresholding power of 6 for proteomic data and 18 for transcriptomic data. The soft thresholding power was determined using the criterion of approximate scale-free topology. Using hierarchical clustering, WGCNA partitions the total set of genes or proteins into distinct, non-overlapping modules labeled by color. In addition, each module corresponds to a module eigengene, which is the weighted average expression profile of the module. Each proteomic module generated by WGCNA was tested for correlation with TGFβ1 treatment. Transcriptomic modules generated by WGCNA were tested for correlation with TGFβ1 treatment and significant proteomic modules. Proteomic module-trait correlations with a Bonferroni corrected P-value less than 0.007 accounting for the number of modules generated by WGCNA (N = 7 modules) were considered statistically significant. Transcriptomic module-trait correlations with a Bonferroni corrected P-value less than 0.0045 accounting for the number of modules generated by WGCNA (N = 11 modules) were considered statistically significant.

### Identification of genes associated with pirfenidone and nintedanib treatment

Genes annotated as associated with pirfenidone and nintedanib treatment were identified using Coremine medical (https://coremine.com/medical/)^48^. Coremine presents results as a network that describes relationship between search terms (pirfenidone and nintedanib) and biological terms (including, but not limited to, gene and proteins terms) discovered through text-mining of the MEDLINE database (i.e. titles and abstracts contained in PubMed)^48^. The strength of the association between search terms and biological terms is based on the number of co-occurrences of both terms in the literature.

### Pathway analysis

We constructed both single gene expression probe/peptide models and network models. Protein set enrichment analysis (PSEA) of single proteins and gene set enrichment analysis (GSEA) of single genes significantly differentially associated with TGFβ1 treatment was performed using the Molecular Signatures Database (MSigDB) v7.0^49^. This included gene set collections comprised of the hallmark gene set (N = 50 gene sets), GO gene sets (N = 9996 gene sets), and curated gene sets (N = 5501 gene sets). In addition, downstream analysis also took advantage of annotations described above. GSEA was also performed on the subset of proteins and genes that were identified as both significant single proteins and single genes significantly differentially associated with TGFβ1 treatment. Additionally, GSEA was performed on genes annotated as associated with pirfenidone only, nintedanib only, and by both pirfenidone and nintedanib within transcriptomic modules significantly correlated with proteomic modules. The Cytoscape EnrichmentMap R package^50^ was used to visualize hub proteins in proteomic modules significantly correlated with TGFβ1 treatment and hub genes in transcriptomic modules significantly correlated with TGFβ1 treatment. Hub proteins were defined as proteins having a kME value greater than 0.90 and hub genes were defined as genes having a kME greater than 0.99. PSEA and GSEA significance was defined as having an FDR-q p-value less than 0.05.

### Downstream interpretation and annotation of WGCNA module members

First, we compiled a list of pathways known to be involved with TGFβ1 signaling if they occurred MSigDB v7.0 along with TGFβ1 (see supplementary Table S7). In addition, drugs with the potential to be repurposed were identified using the Broad Institute CLUE drug repurposing tool (https://clue.io/repurposing-app)^51^. This information was used to annotate genes associated with pirfenidone only, nintedanib only, and by both pirfenidone and nintedanib within transcriptomic modules significantly correlated with proteomic modules and TGFβ1 treatment.

## Supplementary Information

Supplementary Information.

## Data Availability

The transcriptomics data analyzed in the current study (GSE17518) are available through the Gene Expression Omnibus (https://www.ncbi.nlm.nih.gov/geo/). The proteomics data analyzed in the current study are available from the corresponding author on reasonable request.
